# The added value of a mobile application of Community Case Management on referral, re-consultation and hospitalization rates of children aged under 5 years in two districts in Northern Malawi: study protocol for a pragmatic, stepped-wedge cluster-randomized controlled trial

**DOI:** 10.1186/s13063-017-2213-z

**Published:** 2017-10-11

**Authors:** Victoria Hardy, Yvonne O’Connor, Ciara Heavin, Nikolaos Mastellos, Tammy Tran, John O’Donoghue, Annette L. Fitzpatrick, Nicole Ide, Tsung-Shu Joseph Wu, Griphin Baxter Chirambo, Adamson S. Muula, Moffat Nyirenda, Sven Carlsson, Bo Andersson, Matthew Thompson

**Affiliations:** 1 Department of Family Medicine, University of Washington, Seattle, WA 98195-4696 USA; 2 Health Information Systems Research Centre, Cork University Business School, University College Cork, Cork, Ireland; 3 Global eHealth Unit, Department of Primary Care and Public Health, Imperial College London, London, UK; 4 Department of Epidemiology, University of Washington, Seattle, WA USA; 5 Department of Global Health, University of Washington, Seattle, WA USA; 6 Luke International (LIN), Malawi Office, Mzuzu, Malawi; 7 Faculty of Health Sciences, Mzuzu University, Luwinga, Mzuzu, Malawi; 8 Department of Public Health, School of Public Health and Family Medicine, College of Medicine, Cork, Malawi; 9 London School of Hygiene and Tropical Medicine, Keppel St, London, UK; 10 Department of Informatics, Lund Universitet, School of Economics and Management, Lund, Sweden

**Keywords:** Integrated management for childhood illness (IMCI), Community health workers, Child health, Infectious diseases, mHealth, Malawi

## Abstract

**Background:**

There is evidence to suggest that frontline community health workers in Malawi are under-referring children to higher-level facilities. Integrating a digitized version of paper-based methods of Community Case Management (CCM) could strengthen delivery, increasing urgent referral rates and preventing unnecessary re-consultations and hospital admissions. This trial aims to evaluate the added value of the Supporting LIFE electronic Community Case Management Application (SL eCCM App) compared to paper-based CCM on urgent referral, re-consultation and hospitalization rates, in two districts in Northern Malawi.

**Methods/design:**

This is a pragmatic, stepped-wedge cluster-randomized trial assessing the added value of the SL eCCM App on urgent referral, re-consultation and hospitalization rates of children aged 2 months and older to up to 5 years, within 7 days of the index visit. One hundred and two health surveillance assistants (HSAs) were stratified into six clusters based on geographical location, and clusters randomized to the timing of crossover to the intervention using simple, computer-generated randomization. Training workshops were conducted prior to the control (paper-CCM) and intervention (paper-CCM + SL eCCM App) in assigned clusters. Neither participants nor study personnel were blinded to allocation. Outcome measures were determined by abstraction of clinical data from patient records 2 weeks after recruitment. A nested qualitative study explored perceptions of adherence to urgent referral recommendations and a cost evaluation determined the financial and time-related costs to caregivers of subsequent health care utilization. The trial was conducted between July 2016 and February 2017.

**Discussion:**

This is the first large-scale trial evaluating the value of adding a mobile application of CCM to the assessment of children aged under 5 years. The trial will generate evidence on the potential use of mobile health for CCM in Malawi, and more widely in other low- and middle-income countries.

**Trial registration:**

ClinicalTrials.gov, ID: NCT02763345. Registered on 3 May 2016.

**Electronic supplementary material:**

The online version of this article (doi:10.1186/s13063-017-2213-z) contains supplementary material, which is available to authorized users.

## Background

Community Case Management (CCM) is a derivative of the paper-based Integrated Management for Childhood Illness (IMCI) clinical algorithm. The strategy was developed by the World Health Organization (WHO) and United Nations Children’s Fund (UNICEF) to reduce the burden of morbidity and mortality from leading causes of disease (i.e., pneumonia, malaria and diarrhea) among children aged under 5 years in low- and middle-income countries (LMICs) [1–3]. CCM is a widely used intervention for children aged under 5 years and is deployed at village clinics by the largest cadre of community health workers (CHWs) in Malawi, known as health surveillance assistants (HSAs) [4].

Using a stepwise approach, CHWs are guided through a “Sick Child Form,” constituting a series of standardized questions and assessment items to direct clinical management [5]. Depending on the manifestation of “sick” and “danger” signs, which are recorded by hand in a village clinic register, children are given medicine or managed at home. Those children who are severely unwell and outside the scope of CHWs’ practice [6] are urgently referred to the nearest higher-level health facility for more comprehensive clinical management (which can be primary- or secondary-care facilities including health centers, community/rural or district hospitals), with advice given to caregivers about the reason for the referral and the location of the nearest facility [7].

Correct identification of children requiring urgent referral is largely dependent upon CHWs’ fidelity to CCM guidelines, which has been frequently reported as suboptimal [3, 7–10]. Poor completion of relevant CCM assessment items may hinder early recognition of serious illness, reducing urgent referrals to higher-level facilities. Information about CCM-directed urgent referral rates in Malawi is limited. However, assessment of the quality of implementation of the CCM program after national scale-up in 2008 reported that CHWs on average made two urgent referrals (per 1000 children) each month [11]. Given the countries’ annual childhood mortality rates from pneumonia (23%), malaria (14%) and diarrhea (18%) [12], this suggests that many children meeting the classification for urgent referral are not being identified. Under-referral risks repeat consultations [13] for the same illness episode as well as hospitalization of those children with serious illness who are left untreated. But, due to the lack of an integrated and electronic medical record system [14, 15], it is unclear which children are brought back to village clinics because they are still unwell, and which of those admitted to hospital were urgently referred by CHWs at the index visit.

Of equal importance is that children who are urgently referred are actually taken to higher-level facilities (in other words, that the referral is completed) when advised [16, 17]. Failure to follow recommendations risks the development of chronic conditions or acute complications [18]. It is suggested that over 50% of caregivers in sub-Saharan Africa whose children are urgently referred, do not take their child for onward care [16, 19–21]. Prohibitive financial and time-related costs incurred by caregivers travelling large geographical distances to facilities [22], and mistrust in CHW “diagnoses” and treatment decisions [23, 24] have been cited as factors contributing to poor referral completion rates.

Utilizing mobile health (mHealth) technologies (e.g., smartphones, tablets) to deliver CCM could encourage the dispensation of more appropriate treatment recommendations [25] thereby increasing urgent referral rates. Potential improvements may be underpinned by a combination of better control of how the end-user navigates through assessment questions (e.g., built-in validation rules forcing completion of relevant fields with valid data before advancing to the next field/screen) [26]; enhanced CHW motivation linked to the prestige of using advanced technology in a predominantly paper-based health care system; automated presentation of the next-steps reducing reliance on memory [27], and heightened perceived efficacy and caregiver trust in CHWs’ decisions linked to clearer explanations about the child’s condition and recommended treatment [27].

Despite the opportunities that mHealth may afford for circumventing some of the limitations of current paper-based delivery [26], there is a paucity of evidence demonstrating the comparative benefits of deploying digitized versions of CCM on health service utilization and associated patient costs. To address this evidence gap in a pragmatic cluster-randomized trial, we will evaluate the added value of an electronic CCM App (truncated to SL eCCM App) developed as part of the Supporting Low-cost Interventions For disEase control (Supporting LIFE) program. As well as contributing to the broader mHealth literature, we anticipate that trial findings will provide Malawi’s mHealth Working Group with empirical data, which can be used to inform policy regarding the opportunities to Malawi of introducing such interventions for CCM. Our hypothesis is that adding the SL eCCM App to sick child assessments will increase urgent referral rates and reduce re-consultation and hospitalization rates.

The main objectives of this trial were to determine the:

- ▪ Added value of the SL eCCM App (used with paper-CCM) on referral, re-consultations and hospitalization rates among acutely unwell children aged under 5 years, compared with paper-CCM alone
- ▪ Factors influencing caregivers’ decisions to comply with urgent referral recommendations
- ▪ Direct and indirect costs to caregivers presenting to higher-level facilities and/or re-attending village clinics after the index visit when using paper-CCM and the paper-CCM + SL eCCM App

### Previous work justifying this trial

To address repeated calls for bridging the evidence gap regarding mHealth interventions in LMICs [28–30], the Supporting LIFE study team, which consisted of health information systems (HIS) researchers, physicians, epidemiologists and clinical researchers from Imperial College London (ICL), University College Cork (UCC), University of Washington (USA), Lund Universitet (Sweden), Mzuzu University, Luke International (LIN) and the College of Medicine (Malawi), purposely developed the SL eCCM App [31]. Despite CCM being one of the most ubiquitous child health interventions available in LMICs, there have been few attempts to evaluate digitized versions of these guidelines beyond pilot and feasibility studies. Given the variable standards with which CCM is delivered by CHWs, there is a need to identify alternative solutions to enhance quality of health care delivery in the community.

The Supporting LIFE study team conducted a feasibility study between July and September 2015 which explored the acceptability and usability of the SL eCCM App among HSAs in Malawi and the study procedures proposed as part of this trial (the feasibility study, including findings are described in [15] or are forthcoming elsewhere). This trial builds on this previous work.

## Methods/design

### Trial design

**Fig. 1:**
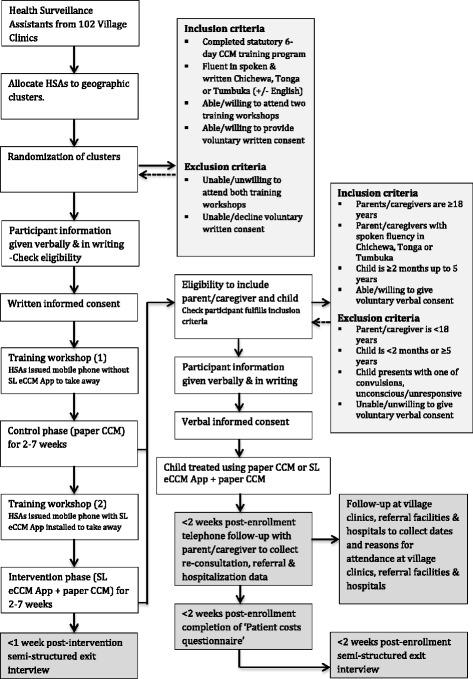
Trial flow diagram

**Fig. 2:**
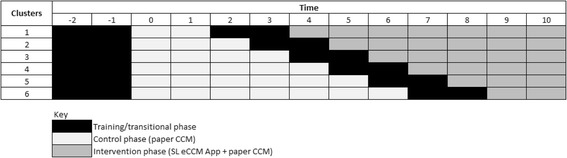
Trial design

**Fig. 3:**
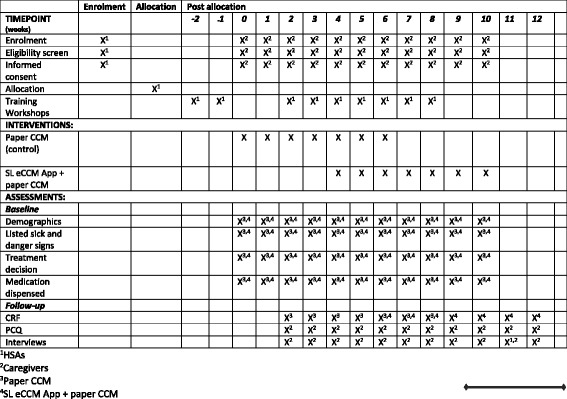
Standard Protocol Items: Recommendations for Interventional Trials (SPIRIT) Figure

This is a pragmatic, stepped-wedge cluster-randomized trial assessing the added value of the SL eCCM App (used with paper-CCM) on referral, re-consultation and hospitalization rates of children aged under 5 years, within 7 days of the index visit. This design was chosen due to insufficient human resources to deliver the intervention to all clusters simultaneously [32] and local advice that withholding the intervention to some clusters may negatively affect participation. We aimed to recruit 102 HSAs and collect data on 8000 children from village clinics across two districts in Northern Malawi between July 2016 and February 2017. HSAs were grouped into six geographic-based clusters. Training workshops were conducted prior to the control (paper-CCM) and intervention (paper-CCM + SL eCCM App) in assigned clusters. Clusters were randomly assigned to the timing of crossover from the control to the intervention [32] for covariate equipoise between phases [33]. A nested qualitative study explored HSA and caregiver perceptions of caregiver adherence to urgent referral recommendations; a cost evaluation investigated the financial and time-related costs to caregivers of seeking subsequent health care for their child after the index visit. Figure 1 shows the trial flow chart; Fig. 2 the trial design (before randomization) and Fig. 3 the Standard Protocol Items: Recommendations for Interventional Trials (SPIRIT) Figure. This protocol adheres to the SPIRIT 2013 Statement for clinical trial protocols (Additional file 1) [34].

## Methods

### Study setting

Malawi is a land-locked country in central southern sub-Saharan Africa [35]. It is one of the poorest nations in the world, ranked 160th out of 182 on the Human Development Index [36]. Although 68% of health care services are government subsidized [18], physical access is limited and inequitable, with only 54% population residing within 5 km of the nearest health facility [37]. Although English has been the official language of Malawi since Independence in 1964 the majority of the population have limited proficiency. Chichewa is the language spoken in Nkhata Bay and Tonga and Tumbuka are spoken in Rumphi District; all of which were provisioned for in this trial. HSAs are posted to hard-to-reach locations where they deliver a mixture of preventative and curative community health programs to a catchment area of approximately 1000 people [4]. They receive 6 days of initial CCM training [5, 6, 11] before implementing CCM to children aged under 5 years from village clinics (operated by one HSA). Village clinics are equipped with rudimentary job aids, which typically include the paper-CCM decision pro forma in English (referred to as the Sick Child Form), a village clinic register for recording patient visits [5], a stopwatch for measuring respiratory rate (RR), and access to basic medications.

### Eligibility criteria

#### Inclusion criteria

Participants were eligible to be included in the trial if they fulfilled the following criteria:

##### Health surveillance assistants

- ▪ Completion of the Ministry of Health’s (MoH’s) 6-day CCM training program
- ▪ Fluent in spoken and written Chichewa, Tonga or Tumbuka (± English)
- ▪ Attendance at both training workshops
- ▪ Voluntary written consent

##### Caregivers

- ▪ Caregivers are aged 18 years and older
- ▪ Caregivers with spoken fluency in Chichewa, Tonga or Tumbuka (it is not anticipated that many will speak English)
- ▪ Child is aged 2 months or older and up to 5 years
- ▪ Voluntary verbal consent

#### Exclusion criteria

Participants fulfilling any of the following criteria were not eligible to take part in this trial:

##### Health surveillance assistants

- ▪ Unable/unwilling to attend both training workshops
- ▪ Unable/decline voluntary written consent

##### Caregivers

- ▪ Caregiver is aged below 18 years
- ▪ Child is aged below 2 months or 5 years and older
- ▪ Child is convulsing, unconscious or unresponsive at presentation
- ▪ Unable/unwilling to give voluntary verbal consent

### The Supporting LIFE electronic Community Case Management Application

**Fig. 4:**
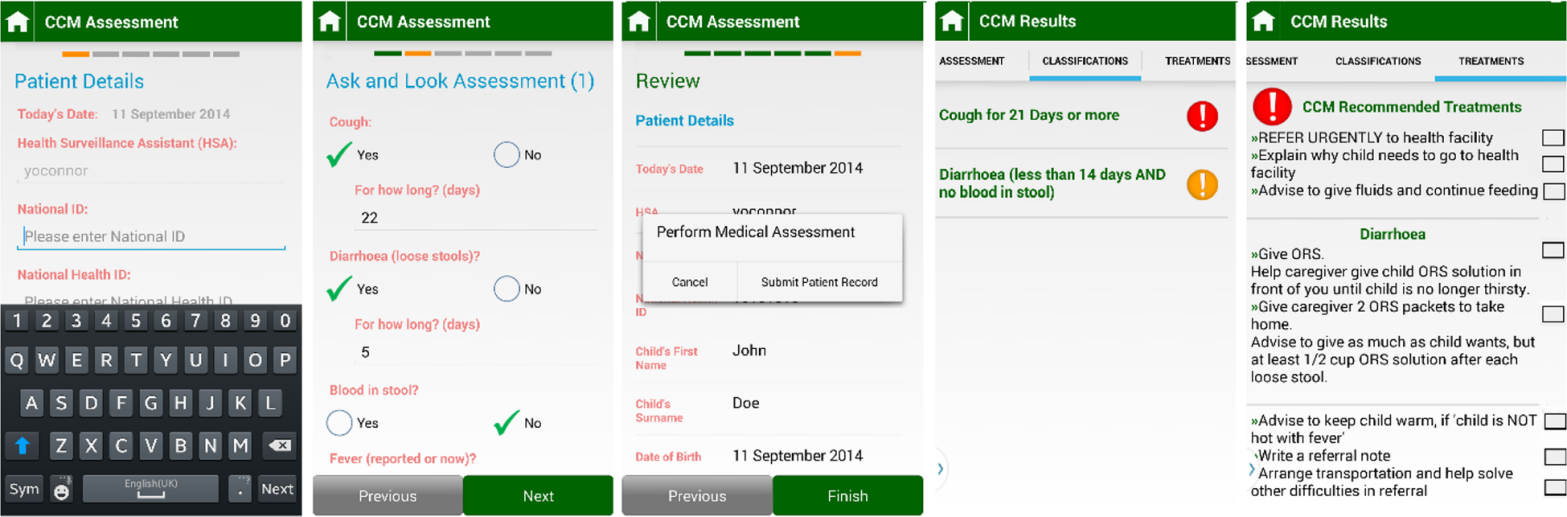
Screenshots of the Supporting LIFE electronic Community Case Management Application (SL eCCM App)

**Fig. 5:**
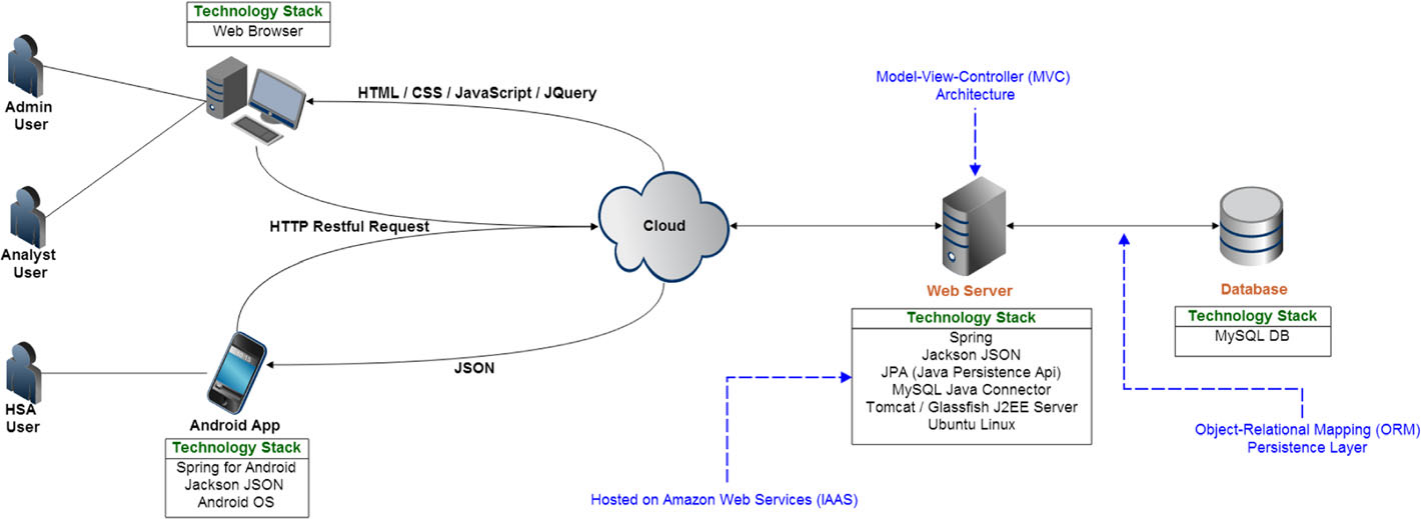
System architecture supporting the Supporting LIFE electronic Community Case Management Application (SL eCCM App)

SL eCCM App is a smartphone App developed for Android OS 3.0 Honeycomb or above; the smartphone selected to run the App for this trial was the HTC Desire 526G. The App is in English and replicates the paper-based CCM decision aid (i.e., the Sick Child Form) in terms of wording and order of presentation of assessment questions (Fig. 4). Patient data is entered via a touch-sensitive dynamic interface. Field validation forced HSAs to complete all required fields prompted by the App’s decision rule and within clinically valid parameters. Current configuration of the SL eCCM App only permits data entry and so previously entered records cannot be retrieved. Included in the App is a countdown tap-screen feature for measuring RR. The number of screen taps within 60 s equates to a child’s RR. Whilst 60 s was the default measurement period for the trial, the App permits the user to select shorter periods (i.e., 15, 30 and 45 s), where a RR equivalent of 60 s is calculated from the time intervals between taps. HSAs were encouraged to use this feature, but could elect to use the stopwatch (as per standard practice). The SL eCCM App uses RESTful web services communicating over JSON to a Cloud-based web server, running on an Amazon Elastic Compute Tomcat instance. The web-server comprises a middle-tier Spring Model-View Controller framework and uses Java Persistence API to communicate to a back-end MySQL database running on an Amazon Relation Database Service instance. My SQL database (referred to as the SL central database) is supported by Amazon Web Services (AWS) Cloud data storage solutions, and has been created to store uploaded patient data from the SL eCCM App (Fig. 5). Patient data is uploaded onto the SL central database when the App is physically synced with the central database. The robustness and fidelity of the App’s clinical algorithm to paper-CCM was tested iteratively over the 4 years of development against a suite of automated tests. It also underwent additional manual checks by members of the study team (which includes a family physician), by creating artificial clinical scenarios and cross-referencing the logic with the Sick Child Form), as well as with 12 HSAs in a feasibility study conducted in Malawi between July and September 2015 [15].

### Outcome measures

#### Main outcomes

- ▪ Urgent referrals to higher-level facilities in the 7 days following the index visit, ascertained from abstraction of patient records 2 weeks after the index visit.*
  - ◦ Caregiver-completed referrals in the 7 days following the index visit, determined from abstraction of patient records (i.e., caregivers who were urgently referred and who presented)
  - ◦ Caregiver self-referrals to primary or secondary health facilities (i.e., parents/caregivers of children not urgently referred at the index visit, but who presented anyway)
- ▪ Re-consultations at village clinics due to deterioration of illness, and hospitalizations in the 7 days following the index visit ascertained from abstraction of patient records 2 weeks after the index visit

*Primary outcome measure used to determine the number of participants needed to power the trial.

#### Other outcomes

- ▪ HSA and caregiver-perceived barriers and facilitators to caregiver compliance with urgent referral recommendations
- ▪ Direct and indirect costs to caregivers of planned and unplanned re-consultations and urgent referrals to any type of health facility (i.e., health centers, community/rural and district hospitals)
- ▪ HSA and caregiver attitudes towards the SL eCCM App and the intervention experience

### Recruitment

Recruitment was coordinated from Luke International Offices in Mzuzu, Mzimba North District. HSAs were identified from village clinics listed on the Demographic and Health Information System 2 (DHIS 2 http://www.dhis2.org/) database (a repository for centralizing health data) that is used widely across Africa [38]. Guidance was sought from district-level Ministry of Health (MoH) officials (e.g., District Health Officer, District Environmental Health Officer, IMCI coordinator) in Rumphi and Nkhata Bay to identify the status of listed village clinics (i.e., whether an HSA is in situ) and how to approach/access HSAs. A reserve of HSAs was identified in case any identified village clinics are inactive, or HSAs do not wish to participate. HSAs were to be invited to participate by the local study team, either in person or over the telephone. Caregivers and their children were identified and enrolled consecutively by HSAs as they presented to village clinics. Based on average HSA recruitment rates during the feasibility study of 10 children per HSA each week, we anticipated that 8000 children would be enrolled into the trial.

## Assignment of interventions

### Randomization, allocation and blinding

HSAs were stratified by geography into six clusters by the local study team and each cluster was given a number between 1 and 6 Using computer-generated simple randomization techniques, each of the six clusters was randomized to the sequence and timing at which they crossed over from the control to the intervention (Fig. 3) [39]. Clusters were randomized once allocated to geographic clusters. Caregivers and their child were allocated to the control or intervention by chance, depending on the time that they presented to village clinics. Due to the nature of the intervention, neither trial participants nor the trial team were able to be blinded to assignments.

### Trial procedures

#### Training workshops

HSAs attended two 1-day training workshops where they learnt how to use the SL eCCM App and became familiarized with study procedures with other HSAs in the same cluster. At the first training workshop HSAs were taught how to operate the smartphone (e.g., turning device on/off; navigation to the SL eCCM App); use the SL eCCM App (e.g., navigate through clinical questions and assessment items; were taught procedures for uploading data onto the central database, using the embedded breathing rate counter) and follow study procedures (i.e., how to recruit and obtain verbal consent from caregivers, and record data in the SL eCCM App and paper-CCM in parallel). At the end of the first training workshop a mobile phone without the SL eCCM App was issued to HSAs, allowing them to become familiar with the device. The App was removed from devices at this time to prevent HSAs using it to treat children during the control phase.

The second workshop was a “refresher” session, conducted 1–2 weeks before each cluster crossed over to the intervention (i.e., paper-CCM + SL eCCM App). The SL eCCM App was downloaded onto the mobile phones assigned to HSAs, ready for the intervention phase. Material covered in the first workshop was also revised. To consolidate learning and encourage compliance with study procedures, clinical vignettes were conducted to simulate study procedures expected to be undertaken by HSAs in the intervention phase.

To ensure a manageable ratio of trainers to HSAs, training workshops were limited to a maximum of 20 HSAs. Training workshops were led by the study team in English, and included a team member fluent in the primary language spoken by participants who could facilitate communication. During the blocked-out time periods allocated to training workshops, HSAs continued to use paper-CCM as per standard practice, but were not able to enroll children during this transitory stage (prevented by removing all study materials from village clinics).

## Data collection

### Baseline

#### Control

Paper-CCM was used to assess and treat children for between 2 and 7 weeks (dependent on clusters’ assigned sequence from randomization), as per standard practice. Facilitated by the Sick Child Form, patient data routinely collected by HSAs through a series of questions and physical examinations was recorded in the village clinic register and included: demographic data (i.e., child and caregiver name, child date of birth, relationship of caregiver to child, physical address); the presence and duration of “sick” signs (i.e., diarrhea for less than 14 days without blood in stool, fever for less than 7 days, red eye for less than 4 days, fast breathing) and “danger” signs (i.e., cough for 21 days or more, diarrhea for 14 days or more, blood in stool, fever lasting for 7 days, convulsions, not able to feed or drink anything, vomits everything, red eye for 4 days or more, red eye with visual problem, chest recession, very sleepy or unconscious, palmar pallor, red/yellow on MUAC tape, swelling of both feet); treatment decision (i.e., urgent referral or treat at home and advise on homecare, including when to return for follow-up) and medication dispensed (e.g., antibiotics, oral rehydration therapy).

#### Intervention

After HSAs in each cluster completed their allocated time in the control and the second training workshop, they transitioned to using the SL eCCM App in addition to paper-based CCM for between 2 and 7 weeks. The same patient data recorded in the control was collected and recorded concurrently in the SL eCCM App and village clinic register. Dual-use and double-data entry were necessary due to functional limitations of the SL eCCM App and because completion of the village clinic register is mandatory for monthly aggregation of cases for country-level disease surveillance purposes. During technical malfunctions (e.g., software bugs, power failures, etc.) that prevented use of the App, or scenarios where caregivers could not be consented, HSAs temporarily reverted to paper-CCM only. HSAs dispensed treatment using recommendations prompted by the SL eCCM App, but could elect to follow recommendations of paper-CCM where they strongly disagreed with the App’s recommendations. Use of the breath count feature to measure RR was encouraged, but HSAs could choose to use the stopwatch (as per standard practice) if they preferred.

### Follow-up

Two weeks following enrollment, caregivers recruited in both the control and intervention phases were contacted by the study team via the mobile telephone number (provided at recruitment), or in person (if they did not provide a number, or were unreachable on the number provided). They were asked by one of the Malawian study team for details of: treatment recommendation given by HSAs at the index visit; re-attendances at village clinics, presentations to higher-level facilities and hospitalizations of their child in the 7 days following the index visit. Since identification of patient records at sites is anticipated to be cumbersome due to poor medical administrative infrastructure [15, 40], information provided by caregivers was used to direct the study team to sites where a record of the patient visit was most likely to be available. Caregiver-reported data will be cross-referenced with data abstracted from patient records to determine the trustworthiness of gathering data from village clinics and higher-level health facilities.

The study team systematically visited participating village clinics and higher-level health facilities within the catchment area of recruiting village clinics to retrospectively obtain the following from patient records: HSAs’ treatment decisions and scheduled follow-up visits with caregivers at the index visit (i.e., urgent referral, treated at home/advice to caregiver); dates of re-attendances at village clinics (including signs/symptoms at presentation and medications administered, if any); dates of attendances and hospitalizations (including diagnoses and medication administered, if any). Follow-up data was recorded on a Case Report Form (CRF).

A subgroup of caregivers, recruited in both the control and intervention phases, who returned to village clinics or who took their child to a higher-level health facility (irrespective of whether an “urgent referral” recommendation was given at the index visit), were asked to complete a Patient Costs Questionnaire (PCQ). The survey was adapted from the Cooking and Pneumonia Study (CAPS) implemented in Malawi [41] and collected sociodemographic details (e.g., level of education, marital status, occupation), financial (e.g., cost of transportation, medication) and time-related (e.g., duration of travel) costs associated with seeking subequent health care for their child. Where practical, survey data was collected from caregivers over the telephone, or, obtained in person in the community with the assistance of the recruiting HSA. Because we anticipated mixed literacy levels among the patient population, the survey was completed either by the caregiver, or by a member of the study team fluent in the local language, who read the questions and scribed on behalf of caregivers. A convenience sample of enrolled HSAs and caregivers from each cluster participated in a semi-structured interview 1–2 weeks after the intervention (HSAs) or recruitment (caregivers), respectively. One-to-one interviews were conducted in person (or depending on location/availability of caregivers, over the telephone) by a member of the study team fluent in the primary language of participants. This interview format was selected due to the influence that cultural factors (e.g., collectivism, gender and sociodemographic inequities) could have on openness of communication in group interviews [42]. Tailored, semi-structured topic guides were used to facilitate interviews and included questions relating to perceptions of the barriers and facilitators to caregiver compliance with urgent referral recommendations and the perceived impact of the SL eCCM App. Topic guides were developed by the study team and were informed by nascent research related to challenges of accessing health care in LMICs, and from findings from focus groups held as part of the feasibility study. All interviews were audio-recorded, lasted between 30 and 40 min, and were held in private. Data collection instruments and/or interview guides can be made available upon request to the corresponding author.

### Sample size

We planned to recruit a total of 102 HSAs. Our sample size estimation is based on change in proportions of the primary outcomes expected pre and post intervention. Assuming an alpha of 0.05, 97 HSAs would provide 80% power for a minimum detectable rate of change between 6 and 7% in referral, re-consultation and hospitalizations (one-sided test). We assumed a 1% urgent referral rate, based on the proportion of children identified as needing to be urgently referred at clinics in a previous study conducted in Malawi [11]. As an urgent referral rate of 8% has been estimated as a reasonable consensus referral rate at clinics in sub-Saharan Africa [43, 44] that are correctly adhering to IMCI, we assumed the addition of the SL eCCM App could improve urgent referral rates close to this estimate; a 6–7% change between the control and intervention was, therefore, a conservative estimate of this assumption. Given the short trial duration and potential of perceived elevated status through smartphone ownership in this setting [45], we did not expect many HSAs to drop out. We believed that recruiting 102 HSAs would be sufficient to accommodate potential dropout, to ensure we achieved our desired sample size. Power calculations were computed using the “Power and Sample Size” option in Stata v. 13 (StataCorp, College Station, TX, USA) [46] which is appropriate for rates, specifically “*longitudinal studies, where the same cases serve as their own controls over time*” [47].

A total of 300 parents/caregivers whose children were referred to a higher-level facility or re-consulted at a village clinic completed the PCQ. It was estimated that approximately 600 children would be eligible for referral to a health care facility assuming: a 1% referral rate among 4000 children in the control group (*n* = 40), a 7–8% referral rate (change 6–7% from the initial 1% referral rate) among the same number of children in the intervention group (*n* = 300) and a 6–7% under-referral rate in the control group (*n* = 260). Considering the challenges in identifying those who were referred but did not present at a health care facility, or those who were not referred but did visit a health care facility or represented at a village clinic, and the possible withdrawal or loss to follow-up of parents/caregivers, it is estimated that at least half (*n* = 300) of the eligible cases will be followed up as part of the cost evaluation.

We also interviewed 15 HSAs and 25 caregivers to explore barriers and facilitators to caregiver compliance with referral recommendations, as well as HSA and caregiver acceptability of the SL eCCM App. Pre-determining the number of participants needed in qualitative research is challenging since the underlying goal of achieving saturation is influenced by factors including interview type (typically one-to-one interviews foster deeper but more narrow exploration of concepts than focus groups, due to absence of exchange of opinions which broaden scope), homogeneity of interviewees, and skill of the researcher (among other things). Guest et al. indicated 12 semi-structured interviews as sufficient to attain saturation [48]. Since our approach is exploratory rather than theoretical, we believed that our target sample size would be sufficient to address our research question and accommodate any heterogeneity among either participant population.

### Data management

To give HSAs an organic user experience, identifiable data was entered into the SL eCCM App. Once the record was saved, patient-identifiable information entered (i.e., child and parent name, physical address) was automatically removed and date of birth was transposed into age (in months). Patient data was held within the SL eCCM App in SQLite database tables until the App was synced with the SL central database, when patient records were automatically removed from the App. De-identified patient data held in database tables within the App was encrypted using secure, 256-bit AES SQLCipher technology. HSAs synced the SL eCCM App with the server from village clinics, or other community locations with a WIFI or data network. Patient records were transmitted by HSAs daily, or as soon as they were able to reach an area with connectivity. Secure Sockets Layer (SSL) technology was used to protect data during transmission from the SL eCCM App to the SL central database, which was managed through a website created to provide an infrastructure for administrative support during the trial (e.g., creating HSA accounts). This website was developed using Java Server Pages (JSP), Bootstrap, JQuery, HTML 5.0 and CSS 3.0. SQLCipher is the technology adopted for provisioning data encryption. SQLCipher is an open-source library that provides transparent, secure, 256-bit AES encryption of SQLite database files. SQLCipher has been adopted as a secure database solution by many commercial and open-source products, making it one of the most popular encrypted database platforms for mobile applications. The SL database is hosted within the AWS data center in Ireland, which is the European base for AWS service provision. User-authentication controls restricted access to the SL eCCM App and SL central database to authorized trial personnel.

The quality of data collection in this trial was contingent upon the completeness and accuracy of patient records at village clinics and higher-level health facilities. To ensure that all available data was abstracted from patient records at these sites, CRF and PCQ Forms were reviewed for completeness by a member of the local study team prior to data entry. De-identified CRF and PCQ data was double entered into the Health Insurance Portability and Accountability Act (HIPAA)-compliant Research Electronic Data Capture (REDCap) system, which was managed by the local trial team in Malawi and monitored remotely by the trial sponsor. REDCap database was set up with double-data entry and validation rules to identify and manage data discrepancies. Data entered into REDCap was reviewed continually during the trial. Missing or inconsistent data was identified and data verification requests generated by the sponsor institution to resolve discrepancies. Individual characteristics of CHWs (e.g., education level) have been associated with enhanced performance in resource-poor settings [49]. Location of village clinics and higher-level health facilities may also influence the quality of medical reporting in patient records and thus the data that is available to be abstracted for this study. HSAs operating in more urban areas may have better access to training and supervision. Urban regions with better infrastructure might have more standardized and organized reporting systems, or conversely, less rigorous procedures because of higher patient volumes. As the factors driving missing data (in addition to attrition of HSAs) in this study risk biasing observed data collected, we cannot assume that data will be missing at random. Therefore, missing data will not be imputed [50]. Upon trial completion, the percentage of missing data between control and intervention phases will be compared. We will use a logistic model to assess whether any covariates significantly predict dropout rates; differential dropout will be assessed. Non-outcome variables will be interpolated to improve multivariate models if needed.

### Confidentiality

Patient records entered into the SL eCCM App during the intervention are identified using a unique, five-digit, study ID number and HSAs are identified from a unique, four-digit number (as pre-populated on completed consent documents). A link between participant ID number and participant names (children and HSAs) is stored in a separate electronic file and will be broken as soon as it is practical to do so. Since a single HSA is typically assigned to each village clinic in Malawi, disclosure of study sites would constitute the release of identifiable data. Therefore, to protect participant’s identity, the list of included village clinics will not be made available. Audio files of interviews will be uploaded and transcriptions stored onto a password-protected computer. Participant identifiers accidentally disclosed during interviews will be removed during transcription and replaced with generic terms (e.g., personal names will be replaced with “a colleague” or “family member”). Participant quotes used in any material resulting from this trial will be protected using their unique ID number. All original study documents (i.e., consent documents, CRFs, PCQs) are stored securely at the local coordinating institution in Malawi (LIN) with access restricted to authorized personnel. After trial closure these trial documents are to be archived locally for 6 years in accordance with the University of Washington’s Human Subjects Division and COMREC’s requirements, and will only be made available under exceptional circumstances (e.g., audit). After verification of data accuracy, electronic datasets will be locked and stored on encrypted institutional networks.

#### Post-trial care

After trial completion, children aged under 5 years presenting to village clinics were to continue treating children aged under 5 years according to standard practice (paper-CCM only).

#### Statistical/analysis methods

Primary outcome data will be evaluated using pairwise comparisons during the control and intervention periods. Analyses will be conducted at the level of the HSA, taking into consideration the number of index visits that occurred during each period, and will include descriptive statistics relating to each outcome (i.e., referral, re-consultation and hospitalization rates); tests of differences by control/intervention period, and multivariate modeling to allow for adjustment of potential confounding factors within each period. Bivariate descriptive statistics will present proportions for categorical variables or means/standard deviations for continuous and categorical data (respectively), for control/intervention periods. Measures of change will be calculated and two-proportion z-tests or chi-square tests will be used to measure unadjusted differences (depending on test assumptions met). We will conduct multivariate analysis using linear regression to evaluate change in outcomes adjusted for composition of children at each HSA by age, gender, symptoms, location (urban/rural) and proximity to the nearest higher-level health facility. For these models, assumptions will be tested, transformations of variables will be made if needed, and a-priori confounders will be evaluated for inclusion in models. Effect modification will be tested by interactions with age and gender. A secondary analysis will be done at the level of the child adjusting for HSA as a cluster variable. Potential confounders and effect modifiers will also be tested in these models. Data analysis and reporting will be conducted with Consolidated Standards of Reporting Trials (CONSORT) [51].

A cost-consequence analysis will be conducted to report the costs and consequences under the control and intervention. All costs of seeking and receiving care for the control and intervention groups will be calculated separately in a disaggregated way at the household level. Direct costs (e.g., out-of-pocket payments and transportation costs), indirect costs (e.g., costs of parent/caregiver productivity loss) and consequences (e.g., hospitalization days) collected through the PCQ will be compared between parents/caregivers enrolled in the control and intervention phases.

Audio-recordings from interviews will be transcribed and translated into English during data collection. It is anticipated that audio-recordings will take some time to be translated and transcribed, making theoretical sampling impractical. Therefore, data synthesis will commence as soon as it is practical to do so. Appropriate qualitative synthesis techniques will be employed, and reporting will adhere to the consolidated Criteria for Reporting Qualitative research (COREQ) [52]. Qualitative data will be triangulated with primary outcomes to contextualize quantitative findings. Qualitative software package NVivo 10 will be used to organize the data.

## Monitoring

### Data Monitoring and Safety Committee (DMSC)

To protect the ongoing rights and safety of participants and the scientific and ethical integrity of the trial, we convened a DMSC. Members of the DMSC were independent of the sponsoring institution and possessed the jurisdiction to periodically review and provide guidance on the handling of adverse events (AEs), protocol deviations and accumulating trial data. Due to the short duration of the trial there were no plans to conduct interim analyses. The DMSC may review and analyze cumulative trial data as requested by its members, at any time during or after the trial. The DMSC possessed the autonomy to recommend protocol modifications, corrective actions, and suspension or termination of the trial if not satisfied that standards of trial conduct were being met.

### Harms

#### Adverse events and serious adverse event reporting

An adverse event (AE) is defined as “any untoward medical occurrence in a trial participant which, having been absent at baseline or if present at baseline, appears to have worsened.” A serious adverse event (SAE) is “any untoward and unexpected medical occurrence or effect that: results in death; is life-threatening (refers to an event during which the participant was at risk of death at the time of event; it does not refer to an event which hypothetically might have caused death had it been more severe); requires hospitalization or prolongation of existing hospitalization, results in persistent or significant disability/incapacity or is a congenital anomaly or birth defect” [53].

AEs were monitored in the first instance by the local trial team and were reported electronically and at bi-weekly team meetings to the sponsor institution. The lead researcher (MT, who is a family physician) and clinical trial program manager (VH) assessed the nature of all reported AEs for causality, expectedness and seriousness. Additional data was requested by the sponsoring institution to determine whether an AE or SAE has occurred. All events will be discussed and, where necessary, management advice sought from the DMSC. Where the incident was deemed to be both related and unexpected, the sponsoring institution informed the Human Subject’s Division at the University of Washington and COMREC within 24 h of receiving notification of the AE.

We expected some children presenting to village clinics to be seriously ill, but we did not anticipate AEs resulting from the use of the SL eCCM App, or from study procedures. Multiple precautionary measures were put in place to prioritize the welfare of participants and maintain standards of health care. Specifically, the SL eCCM App is used as an adjunct to standard care and HSAs possess the autonomy to defer to paper-CCM in the event of disagreement with treatment recommendations or technical issues preventing use. Additionally, children perceived to have life-threateningly illness at presentation were not eligible for enrollment, and the use of the SL eCCM App could be aborted by HSAs at any time and for any reason. The primary concern associated with this trial was the additional workload that double assessment and data entry would impose on HSAs and the resultant increased time taken to assess children, which could negatively impact compliance with study procedures. The study team periodically telephoned HSAs and conducted site visits to ensure proper execution of study procedures and monitor HSAs’ continued willingness to participate. We simplified the processes as much as is possible without compromising participant safety or data quality. These study procedures were successfully implemented in a previous feasibility study, with no deviation from the procedures suspected or found.

### Sponsorship and auditing

The University of Washington was the trial sponsor and was responsible for overseeing all aspects related to the management and conduct of the trial. Responsibilities for trial activities were delegated to collaborating institutions. The trial is open to inspection and audit by the University of Washington as the sponsoring institution, and the Malawi College of Medicine Research Ethics Committee (COMREC), responsible for local oversight of this trial.

### Publication policy and dissemination

The full statistical analysis plan will be made publicly available prior to statistical analysis. The final de-identified dataset will be made available on a public repository. Results from this trial will be presented in aggregate and published in peer-reviewed journals, and in accordance with the Supporting LIFE publication policy. There are no plans to use professional writers for any of the research output from this trial. The authors will declare on all manuscripts, websites, press releases and any other communications materials that the project has received funding from the European Union’s Seventh Framework Program for research, technological development and demonstration under grant agreement number 305292, in acknowledgement of the trial funding body.

## Discussion

To the authors’ knowledge the Supporting LIFE trial is the first large-scale trial evaluating the added value of an electronic version of CCM compared to paper-CCM alone. This trial will provide important data regarding the impact of digitizing CCM on HSA urgent referral practices and factors influencing health service utilization in Malawi. The outcome of referral rate provides a proxy quality indicator: higher urgent referral rates and reduced re-consultation and hospitalization rates compared to paper-CCM may suggest that integrating digitized versions of CCM to the clinical workup improves standards of CCM delivery. Our findings may also be used by the mHealth Working Group in Malawi and decision-makers in other LMICs to better determine the benefit to health care of introducing similar mHealth CCM tools. More broadly, this trial is expected to inform the design and conduct of future evaluations of mHealth technologies for CCM.

Several obstacles influenced how this trial was designed. Firstly, due to a mixture of financial, human resource and time constraints, we were unable to incorporate some the appendages of CCM into the SL eCCM App (i.e., vaccination record) as well as the capability to retrieve previous records of patient visits to review. Secondly, it is a requirement of the Malawian MoH for a physical record of every patient visit to be retained at village clinics. Without integration of the SL eCCM App with the local health information systems, electronic data submission to the MoH was not feasible. Therefore, evaluating the standalone impact of the SL eCCM App was not possible, thus limiting causality between intervention and outcome. Thirdly, the lack of electronic and integrated medical records across health facilities makes corroborating attendances at higher-level health facilities cumbersome. Therefore, a 7-day follow-up period was chosen to balance the follow-up window against the workload and likelihood of being able to locate patient records of at sites. Data of children in developed countries shows that symptoms for respiratory infections can persist for up to 21 days, (mean duration between 4 and 16 days) [54], meaning that some presentations related to the same illness episode may be missed. Similarly, excluding the most severely ill children (i.e., those who are convulsing, unconscious/unresponsive at presentation) may result in under-representation of hospitalization rates. Because of the added time that trial procedures were anticipated to impose on participants, we wanted to ensure that prompt management of these children was prioritized in scenarios where the child’s condition could become rapidly life-threatening. We believe that focusing on outcomes arising immediately after baseline provides a better reflection of the quality of CCM delivery at initial contact.

## Trial status

This trial opened to recruitment on 24 October 2016 and collected follow-up data by 3 February 2017.

## Additional files

Additional file 1: SPIRIT Checklist. List of completed items addressed in this manuscript. (PDF 301 kb)

Additional file 2: Health Surveillance Assistant Recruitment Statement. Written statement to recruit Health Surveillance Assistants to the trial, read by trial personnel. (PDF 202 kb)

Additional file 3: Parent/Caregiver Recruitment Statement (Control). Written statement read by Health Surveillance Assistants to recruit parents/caregivers to the trial in the control phase. (PDF 201 kb)

Additional file 4: Parent/Caregiver Recruitment Statement (Intervention). Written statement read by Health Surveillance Assistants to recruit parents/caregivers to the trial in the intervention phase. (PDF 202 kb)

Additional file 5: Health Surveillance Assistant Consent Form. Document for obtaining written informed consent from Health Surveillance Assistants. (PDF 346 kb)

Additional file 6: Parent/Caregiver Information Statement (Control). Document for obtaining verbal consent from parents/caregivers during the control phase. (PDF 376 kb)

Additional file 7: Parent/Caregiver Information Statement (Intervention). Document for obtaining verbal consent from parents/caregivers during the intervention phase. (PDF 340 kb)

## Abbreviations

AE: Adverse event
AWS: Amazon Web Services
CAPS: Cooking and Pneumonia Study
CCM: Community Case Management
CHW: Community health worker
COMREC: College of Medicine Research Ethics Committee
CRF: Case Report Form
DHIS 2: Demographic and Health Management Information System 2
DMSC: Data Monitoring and Safety Committee
HIPAA: Health Insurance Portability and Accountability Act
HSAs: Health surveillance assistants
IMCI: Integrated Management of Childhood Illness
IRB: Institutional Review Board
LMIC: Low- and middle-income countries
MoH: Ministry of Health
PCQ: Patient Costs Questionnaire
PDA: Personal Digital Assistant
REC: Research Ethics Committee
REDCap: Research and Electronic Data Capture system
SAE: Serious adverse event
SL eCCM App: Supporting LIFE electronic Community Case Management Application
SSL: Secure Sockets Layer
UNICEF: United Nation Children’s Fund
WHO: World Health Organization
