# Supplementary material for: The added value of a mobile application of Community Case Management on referral, re-consultation and hospitalization rates of children aged under 5 years in two districts in Northern Malawi: study protocol for a pragmatic, stepped-wedge cluster-randomized controlled trial
Source: Trials. 2017 Oct 11;18:475. doi: 10.1186/s13063-017-2213-z (PMC5637321; doi:10.1186/s13063-017-2213-z)
Supplement: Supplementary file 2 — Health Surveillance Assistant Recruitment Statement. Written statement to recruit Health Surveillance Assistants to the trial, read by trial personnel. (PDF 202 kb) [file 13063_2017_2213_MOESM2_ESM.pdf]

Supporting LIFE

RECRUITMENT STATEMENT
Health Surveillance Assistant

Study title: The added value of a mobile application of Community Case Management on Under-5 re-consultation, referral and hospitalization rates in Malawi: a pragmatic stepped-wedge cluster randomized trial

Lead Researcher
Matthew Thompson, Department of family medicine, University of Washington
Email: mjt@uw.edu

Subject contact person
Winnie Mkandwire, Luke International (LIN), Malawi
Email: winniemkandawire@gmail.com Tel: +265 994 767 798

My name is [name]. I am from [Luke International (LIN)/Mzuzu University] and I would like to invite you to take part in a research study that I am conducting with universities in Europe and America. We are investigating how helpful a mobile phone version of Community Case Management (CCM) is for identifying which children need to be sent to a referral facility. You are being invited to take part because you are a Health Surveillance Assistant (HSA) and you use CCM to help you assess and treat sick children.

If you decide to take part you will be asked to attend two training workshops with other HSAs to learn how to use the mobile version of CCM. Each training workshop will last a full day. The first workshop will be held within the next 2 weeks. After the first workshop you will be asked to continue to use paper CCM to assess and treat sick children, as you would do normally for a minimum of 2 weeks and a maximum of 7 weeks, determined by the study team. You will then be asked to attend a second training workshop before you are given the mobile phone of CCM for you to test at your clinic for a minimum of 2 weeks and a maximum of 7 weeks, determined by the study team. During testing, you will be asked to record information about a child's illness in both the mobile version of CCM and the village clinic register. Because we would like you to record information from parents/caregivers that you would not usually collect (i.e. children's date of birth, mobile phone number) and use a new device to help you assess and treat sick children, you will be asked to get verbal permission from each parent/caregiver beforehand.

The study team might invite you to take part in an interview to ask you some questions about the mobile version of CCM. If you are invited, the interview will last between 30 and 40 minutes and will be voice-recorded.

Taking part in this study is your choice. If you do not want to take part you can say 'No'. If you think you would like to take part, please read the study Consent Form.

Do you have any questions?
Thank you for your time
