# Supplementary material for: The added value of a mobile application of Community Case Management on referral, re-consultation and hospitalization rates of children aged under 5 years in two districts in Northern Malawi: study protocol for a pragmatic, stepped-wedge cluster-randomized controlled trial
Source: Trials. 2017 Oct 11;18:475. doi: 10.1186/s13063-017-2213-z (PMC5637321; doi:10.1186/s13063-017-2213-z)
Supplement: Supplementary file 3 — Parent/Caregiver Recruitment Statement (Control). Written statement read by Health Surveillance Assistants to recruit parents/caregivers to the trial in the control phase. (PDF 201 kb) [file 13063_2017_2213_MOESM3_ESM.pdf]

Supporting LIFE

RECRUITMENT STATEMENT
Parent/caregiver (Control)

Study title: The added value of a mobile application of Community Case Management on Under-5 re-consultation, referral and hospitalization rates in Malawi: a pragmatic stepped-wedge cluster randomized trial

Lead Researcher
Matthew Thompson, Department of family medicine, University of Washington
Email: mjt@uw.edu

Subject contact person
Winnie Mkandwire, Luke International Norway, Malawi
Email: winniemkandwire@gmail.com Tel: +265 994 767 798

I would like to invite you to take part in a research study that I am helping Mzuzu University, Luke International Norway and universities in America and Europe with. We are investigating how helpful the Community Case Management tool is for identifying which children need to be sent to a referral facility. I am inviting you to take part in this study because you think your child is sick.

If you decide to take part, I will use the Community Case Management tool to help me assess and treat your child, as I would do normally. I will also ask you to give me some additional information about you and your child. This will include a mobile phone number if you have one, which the study team will use to contact you.

The study team will telephone you in approximately 1 to 2 weeks' time. The reason they will telephone you is to find out if you took your child to a referral facility, hospital or re-visited a village clinic. A member of the study team will look at healthcare records at the health facilities you mention to record the dates of your child's visits.

The study team might also contact you to invite you to take part in an anonymous survey. If you are invited, the interview will last between 30 and 40 minutes and will be voice-recorded. The survey will ask you some brief questions about any costs (related to time and money) associated with taking your child for additional treatment at health facilities.

Taking part in this study is your choice. If you do not want to take part you can say 'No'. If you think you would like to take part, please read the study information statement.

Do you have any questions? If you would like to take part please tell me and I will collect your name, telephone number and some other details from you.

Thank you for your time
