# Supplementary material for: The added value of a mobile application of Community Case Management on referral, re-consultation and hospitalization rates of children aged under 5 years in two districts in Northern Malawi: study protocol for a pragmatic, stepped-wedge cluster-randomized controlled trial
Source: Trials. 2017 Oct 11;18:475. doi: 10.1186/s13063-017-2213-z (PMC5637321; doi:10.1186/s13063-017-2213-z)
Supplement: Supplementary file 5 — Health Surveillance Assistant Consent Form. Document for obtaining written informed consent from Health Surveillance Assistants. (PDF 346 kb) [file 13063_2017_2213_MOESM5_ESM.pdf]

Supporting LIFE

CONSENT FORM

Health Surveillance Assistant

Study ID

Study title: The added value of a mobile application of Community Case Management on Under-5 re-consultation, referral and hospitalization rates in Malawi: a pragmatic stepped-wedge cluster randomized trial

Lead Researcher
Matthew Thompson, Department of family medicine, University of Washington, USA
Email: mjt@uw.edu

Subject contact person
Winnie Mkandwire, Luke International (LIN), Malawi
Email: winniemkandwire@gmail.com Tel: +265 994 767 798

INVITATION TO PARTICIPATE

We would like to invite you to take part in a research study because you are a Health Surveillance Assistant and you treat sick children using Community Case Management (CCM). The purpose of this form is to give you the information you need to help you decide whether or not you would like to take part in this study. Please read the form carefully. If there is anything you do not understand, please ask a member of the study team to explain. When your questions have been answered, you can decide if you want to be in the study or not. If you do not wish to participate, you are free to say no at any time.

PURPOSE OF THE STUDY

Many children in Malawi get illnesses such as pneumonia and malaria. Health Surveillance Assistants (HSAs) use paper-based CCM guidelines to help them treat sick children. We would like to find out how helpful a mobile phone version of CCM (called the SL eCCM App) is for identifying which children need to be sent to referral facilities, in comparison to paper CCM. We would also like to determine if and when children attend referral facilities, re-visit village clinics or are hospitalized, and the reasons why they attend or do not attend referral facilities when they are sick.

STUDY PROCEDURES

If you decide to take part you will be asked to:

Use paper CCM

Use paper CCM for between 2-7 weeks to assess and treat sick children as you would normally. You will be asked to get verbal permission from each parent/caregiver for you to record some additional information about them and their child (i.e. date of birth and telephone number) on a form similar to this one.

Attend two training workshops

Attend two 1-day training workshops with other HSAs. This is because the SL eCCM App is new, so it is important to learn how to use it properly. Study procedures will also be explained to you during these workshops. The first workshop will be held in the next 1 to 2-weeks and the second 1 to 2-weeks before you test the SL eCCM App.

Test SL eCCM App

You will use the SL eCCM App to help you assess and treat sick children at your village clinic. You will be asked to get verbal permission from each parent/caregiver to use the SL eCCM App, and also for you to record some additional information about them and their child (i.e. telephone number and date of birth). This information will be recorded on a form similar to this one.

A mobile phone with the SL eCCM App will be given to you for a minimum of 2 weeks and a maximum of 7 weeks, decided by the study team. To reliably test how useful the SL eCCM App is, we need to ask HSAs to test it for different lengths of time. During this testing time, you should use the SL eCCM App in addition to paper CCM to assess and treat as many sick children as possible. You

CONSENT FORM

Health Surveillance Assistant

should record information about a child's illness in **both** the SL eCCM App and village clinic register as you complete each assessment question. You should follow treatment recommendations given by the SL eCCM App as much as possible, even if the recommended treatment is different between the SL eCCM App and the paper CCM. However, if you ever disagree with the treatment recommendations given by the SL eCCM App, then you should follow the recommendations from the paper CCM. You should not use the SL eCCM App if a parent/caregiver does not give permission for it to be used, or the phone stops working. If this happens you should use paper CCM instead and record the sick child visit in the village clinic register like you would normally.

Allow the study team to review village clinic records
Allow the study team to review the village clinic register, and record the dates children enrolled into the study were seen again at your village clinic.

Additionally, you might be asked to:
Take part in an interview
The study team might invite you to take part in an interview, no more than a week after testing the SL eCCM App. If you are invited, you will be asked for your opinions of the SL eCCM App and your thoughts on what influences parent/caregivers to follow the urgent referral advice you give. The interview will last between 30-40 minutes and will be voice recorded (with your permission).

DURATION OF INVOLVEMENT
If you agree to take part in this study, you will be involved for a maximum of 6-months. This is from the time you sign this form until the time the study team has finished collecting dates children re-attended at your village clinic, from the village clinic register.

REIMBURSEMENT
You will be reimbursed for any costs incurred travelling to the training workshop and interviews. Lunch will be provided each day of the training workshop. At the end of the research study, and upon adherence to the study procedures and instructions given by the research team, the mobile phone will be given to you to keep. The SL eCCM App will be removed from the phone. This is because we only have permission to use the SL eCCM App for research purposes.

RISKS, STRESS, OR DISCOMFORT
It may take more time than normal to assess and treat sick children at your village clinic. This is because we need to ask you to get verbal permission from each parent/caregiver for you to record some additional information and you will be entering information into both the SL eCCM App and village clinic register. If at any time you think using the device is negatively affecting your ability to do your job or a child's care, you should stop using it immediately and continue to treat the child using paper CCM, as you normally would. If you are invited to take part in an interview, you may feel uncomfortable or self-conscious sharing your thoughts because the interview is being recorded.

ALTERNATIVES TO TAKING PART IN THIS STUDY
Taking part in this study is your choice. You may refuse to participate and are free to leave the study at any time without giving a reason, but you should tell the study contact person. Refusal or withdrawal will not result in a loss of benefits to which you are otherwise entitled. If you withdraw from the study, we will keep information collected from you to help us understand how helpful the SL eCCM App is to HSAs, unless you do not want us to do so, in which case your information will be deleted.

BENEFITS OF THE STUDY
There are unlikely to be any direct benefits to you. Your involvement will help us understand if it is possible to use mobile phones to help assess and treat sick children in future. Upon completion of

CONSENT FORM

Health Surveillance Assistant

the study, you may be eligible to keep the mobile phone, which might be used to help HSAs perform future duties.

CONFIDENTIALITY

Information collected from you will be coded so you cannot be identified. We will keep a link between your name and the code on a password-protected computer. A copy of your study records will be stored on the computer system, but will not be linked to your name. Access to the computer system will be limited to authorized members of the study team with passwords. If we publish the results of this study, we will not use your name. Representatives from the College of Medicine Research Ethics Committee (COMREC) in Malawi or the University of Washington in the USA can sometimes review studies to make sure they are being done safely and legally. If this happens, the information collected from you during this study may be examined. The reviewers will not be able to identify you. The study records will not be used to put you at legal risk of harm.

SPONSOR AND FUNDING

The University of Washington (USA) is the sponsor of this study. The study has received funding from the European Union's Seventh Framework Programme (FP7) under grant agreement number 305292.

STUDY CONTACT

If you would like to ask questions or you decide you no longer wish to take part in the study or you have been harmed by participation in this study, you should **contact Winnie Mkandwire** at **winniemkandwire@gmail.com (Tel: +265 994 767 798).**

To be completed by the Health Surveillance Assistant:

I have read the above information and it has been explained to me. I volunteer to take part in this study. I have had a chance to ask questions. If I have questions about the research or if participating in this study has harmed me, I can contact one of the researchers listed on the first page of this form. If I have questions about my rights as a research subject, I can call the COMREC secretariat on +265 (0) 1 871 900. I will receive a copy of this consent form. I voluntarily agree to take part in this study.

PRINTED NAME OF PARTICIPANT[1] (Please print)	SIGNATURE OF PARTICIPANT[2]	DATE[3]

To be completed by research personnel taking consent:

NAME[4] (Please print)	SIGNATURE[5]	DATE[6]
