# Supplementary material for: The added value of a mobile application of Community Case Management on referral, re-consultation and hospitalization rates of children aged under 5 years in two districts in Northern Malawi: study protocol for a pragmatic, stepped-wedge cluster-randomized controlled trial
Source: Trials. 2017 Oct 11;18:475. doi: 10.1186/s13063-017-2213-z (PMC5637321; doi:10.1186/s13063-017-2213-z)
Supplement: Supplementary file 7 — Parent/Caregiver Information Statement (Intervention). Document for obtaining verbal consent from parents/caregivers during the intervention phase. (PDF 340 kb) [file 13063_2017_2213_MOESM7_ESM.pdf]

INFORMATION STATEMENT

Parents/caregivers: Intervention

Study ID

Study title: The added value of a mobile application of Community Case Management on Under-5 re-consultation, referral and hospitalization rates in Malawi: a pragmatic stepped-wedge cluster randomized trial

Lead Researcher
Matthew Thompson, Department of family medicine, University of Washington, USA
Email: mjt@uw.edu

Subject contact person
Winnie Mkandawire, Luke International (LIN), Malawi
Email: winniemkandawire@gmail.com Tel: +265 994 767 798

Researchers' statement

On behalf of the study team, I would like to invite you to take part in a research study. The purpose of this form is to help you decide whether or not you would like to take part. Please read the form carefully. If there is anything you do not understand, please ask me to explain. When your questions have been answered, you can decide if you want to be in the study or not. If you do not wish to take part, you are free to say no.

PURPOSE OF THE STUDY

In Malawi, many children who are sick get assessed by Health Surveillance Assistants (HSAs) using a paper-based Community Case Management tool. The study team would like to find out how helpful a mobile phone version of Community Case Management is for identifying which children need urgent care. They would also like to know which children attend referral facilities, re-visit village clinics or are hospitalized, and the reasons why children attend or do not attend referral facilities when they are sick.

STUDY PROCEDURES

If you decide to take part I will ask you some questions about your child's illness using a mobile phone to help me. I will record the information you give me in the mobile phone and also in the village clinic register. I will also ask you to give me some additional information about you and your child. This will include a mobile phone number if you have one, which the study team will use to contact you. I will record this information at the bottom of this form.

The study team will telephone you in approximately 1 to 2 weeks' time. The reason they would like to telephone you is to find out if and when your child was taken to a referral facility, hospital or re-visited a village clinic. A member of the study team will look at records at the referral facilities, hospitals and village clinics you mention to record the dates of your child's visits.

You might also be asked to:

The study team might also contact you to invite you to take part in an interview and/or a survey, at a later date. If you are invited to be interviewed, you will be asked some questions about your opinions of the mobile phone and the reasons why you take your child for treatment at referral facilities and village clinics. The interview will last between 30 and 40 minutes and will be voice recorded (with your permission). The survey will ask you some questions about yourself and any costs you experience (such as time or money) taking your child to a health facility for additional treatment. The interview and/or survey will be conducted either by telephone or in person at a location convenient to you and will take a maximum of 20 minutes to complete.

DURATION OF INVOLVEMENT

If you agree to take part in this study, you and your child will be involved for a maximum of 4-months. This includes your visit to the village clinic today, until the time the study team has contacted you or finished collecting the dates your child visited referral facilities and village clinics.

RISKS, STRESS, OR DISCOMFORT

INFORMATION STATEMENT

Parents/caregivers: Intervention

It may take more time than normal for your child to be treated today. This is because the mobile phone version of Community Case Management is new, so I need to ask your permission for it to be used to assess and treat your child. I also need to record the information you give me twice. If at any time you do not want the mobile phone to be used, you should tell me and I will stop using it immediately. I will assess your child using paper Community Case Management, as normal. If you are invited by the study team to take part in an interview, you may feel uncomfortable or self-conscious sharing your thoughts because the interview is being recorded.

BENEFITS OF THE STUDY

There are unlikely to be any benefits to you or the child, other than the benefits you would normally receive when visiting a village clinic. Your involvement will help us understand if it is possible to use mobile phones to assess and treat sick children in future and the reasons why children attend or do not attend referral facilities and village clinics when they are sick.

CONFIDENTIALITY

Information collected from you will be coded so you cannot be identified. A link between your name and the code will be kept on a password-protected computer. A copy of your study records will be stored on the computer system, but will not be linked to your name. Access to the computer system will be limited to authorized members of the study team with passwords. If we publish the results of this study, your name will not be used. Representatives from the College of Medicine Research Ethics Committee (Malawi) or the University of Washington (USA) can sometimes review studies to make sure they are being done safely and legally. If this happens, information collected from you during this study may be examined. The reviewers will not be able to identify you. Study records will not be used to put you at legal risk of harm.

SPONSOR AND FUNDING

The University Washington (USA) is the sponsor of this study. The study has received funding from the European Union's Seventh Framework Programme (FP7) under grant agreement number 305292.

OTHER INFORMATION

Taking part in this study is your choice. If you decide not to take part, you and your child will receive the same standard of care you have always received.

STUDY CONTACT

If you have questions about this research or you decide you no longer wish to take part in the study or you have been harmed by participation in this study, you should **contact Winnie Mkandawire** at **winniemkandawire@gmail.com (Tel: +265 994 767 798)**

To be completed by the Health Surveillance Assistant:

NAME OF CHILD[1]	DATE OF BIRTH[2]	GENDER[3]	DATE[4]
NAME OF PARENT/CAREGIVER[5]	TELEPHONE NUMBER[6]	NAME OF VILLAGE CLINIC[7]	
